# Surveying the Genetic Design Space for Transcription Factor-Based Metabolite Biosensors: Synthetic Gamma-Aminobutyric Acid and Propionate Biosensors in *E. coli* Nissle 1917

**DOI:** 10.3389/fbioe.2022.938056

**Published:** 2022-08-25

**Authors:** Matthew Lebovich, Lauren B. Andrews

**Affiliations:** ^1^ Department of Chemical Engineering, University of Massachusetts Amherst, Amherst, MA, United States; ^2^ Biotechnology Training Program, University of Massachusetts Amherst, Amherst, MA, United States; ^3^ Molecular and Cellular Biology Graduate, Program University of Massachusetts Amherst, Amherst, MA, United States

**Keywords:** genetically encoded biosensor, whole cell biosensor, living therapeutics, gut microbial metabolites, depression, neurotransmitter, gamma-aminobutyric acid (GABA), propionate

## Abstract

Engineered probiotic bacteria have been proposed as a next-generation strategy for noninvasively detecting biomarkers in the gastrointestinal tract and interrogating the gut-brain axis. A major challenge impeding the implementation of this strategy has been the difficulty to engineer the necessary whole-cell biosensors. Creation of transcription factor-based biosensors in a clinically-relevant strain often requires significant tuning of the genetic parts and gene expression to achieve the dynamic range and sensitivity required. Here, we propose an approach to efficiently engineer transcription-factor based metabolite biosensors that uses a design prototyping construct to quickly assay the gene expression design space and identify an optimal genetic design. We demonstrate this approach using the probiotic bacterium *Escherichia coli* Nissle 1917 (EcN) and two neuroactive gut metabolites: the neurotransmitter gamma-aminobutyric acid (GABA) and the short-chain fatty acid propionate. The EcN propionate sensor, utilizing the PrpR transcriptional activator from *E. coli*, has a large 59-fold dynamic range and >500-fold increased sensitivity that matches biologically-relevant concentrations*.* Our EcN GABA biosensor uses the GabR transcriptional repressor from *Bacillus subtilis* and a synthetic GabR-regulated promoter created in this study. This work reports the first known synthetic microbial whole-cell biosensor for GABA, which has an observed 138-fold activation in EcN at biologically-relevant concentrations. Using this rapid design prototyping approach, we engineer highly functional biosensors for specified *in vivo* metabolite concentrations that achieve a large dynamic range and high output promoter activity upon activation. This strategy may be broadly useful for accelerating the engineering of metabolite biosensors for living diagnostics and therapeutics.

## Introduction

The probiotic bacterium *Escherichia coli* Nissle 1917 (EcN) has recently been engineered for use as a therapeutic microorganism to sense and respond to disease biomarkers (Daeffler et al., 2017; Riglar et al., 2017; McKay et al., 2018; Lynch et al., 2022) and treat diseases in the gastrointestinal tract, such as metabolic disease (Isabella et al., 2017; Isabella et al., 2018) and inflammatory bowel disease (Praveschotinunt et al., 2019; Yan et al., 2021). The EcN strain has been used in a multitude of clinical trials (Sonnenborn and Schulze 2009; Ou et al., 2016; Sonnenborn 2016) and has the generally recognized as safe designation from the U.S. FDA, making it a suitable choice to use as an engineered living diagnostic or therapeutic bacterium. In order to use engineered probiotic bacteria in this way, the cells must be genetically programmed to detect the specified biochemical signals in the gastrointestinal tract at the biologically relevant concentrations found in the gut. This requires a metabolite-responsive biosensor to be engineered that has the necessary sensitivity to detect the metabolite. Additionally, the sensor must have sufficiently low leakiness, large dynamic range, high selectivity, and high activated promoter strength to be interconnected with the desired transcriptional regulatory network or gene expression output.

Several different strategies have been used to engineer metabolite biosensors that are comprised of a metabolite-responsive transcriptional regulator and its corresponding cognate promoter. For *in situ* sensing of a metabolite, we are constrained by the metabolite’s concentration in the system, and therefore, the ligand concentration cannot be adjusted to tune the sensor performance, unlike for other applications of whole cell biosensors. Using directed evolution or rational engineering, the attributes of sensors have been improved by mutating the sequence of the sensor output promoter, which can alter its binding affinity to the cognate regulator or RNA polymerase (Lutz and Bujard 1997; Daeffler et al., 2017; Chen et al., 2018; Chen et al., 2019). The coding sequences of regulators have also been mutated to improve sensor performance, such as by altering their ligand binding affinity (Lee et al., 2007; McCready et al., 2019; Meyer et al., 2019). Lastly, another common strategy that has been used to tune sensors is to optimize the expression of the regulator by changing the promoter or ribosome binding site (RBS) controlling it (Brophy and Voigt 2014; Daeffler et al., 2017; Xiao et al., 2017; Meyer et al., 2019; Wang et al., 2019; Ding et al., 2020). However, as the DNA elements of a sensor are altered, there are often tradeoffs in the sensor’s attributes and many properties affected simultaneously, such as leakiness, dynamic range, maximum promoter strength, sensitivity, specificity, and toxicity. This makes it extremely challenging to predict the optimal genetic design. Moreover, when an engineered sensor is transferred to a different host organism, or even a closely-related strain, the sensor’s performance is often affected, and additional tuning is required (Chen and Steele 2005). All these approaches are typically labor-intensive to construct libraries of sensor variants and perform the screening or selections required.

In this work, we present an alternative rapid prototyping strategy to engineer and optimize metabolite biosensors in non-model organisms with minimal DNA assembly required. Instead of constructing a library of sensor variants, we construct a single sensor prototype design to systematically survey the gene expression space for the regulator and identify one final genetic design with improved sensor performance. For this work, we selected two metabolites found in the human gastrointestinal tract to test this approach. Propionate is a highly abundant microbially-derived short-chain fatty acid in the colon (Cummings et al., 1987) that has been suggested to affect many organs (Bindels et al., 2012; Tong et al., 2016; Bartolomaeus et al., 2019), including the brain (Byrne et al., 2016; Hoyles et al., 2018), and play a role in neurological conditions including depression, Alzheimer’s and Parkinson’s diseases, and autism (Wang et al., 2012; Unger et al., 2016; Ho et al., 2018; Müller et al., 2021). In *E. coli*, propionate is converted to 2-methylcitrate and activates the P_PrpB_ promoter via the PrpR transcriptional regulator (Palacios and Escalante-Semerena 2004; Lee and Keasling 2005)*.* The metabolite gamma-aminobutyric acid (GABA) is a human neurotransmitter produced by some bacteria found in the gut (Strandwitz et al., 2019) and has been shown to affect mood and sleep disorders (Sigel and Steinmann 2012; Diez-Gutiérrez et al., 2020), as well as neurological pathologies including epilepsy and depression (Kalueff and Nutt 2007; Diez-Gutiérrez et al., 2020; Ding M. et al., 2021). In *Bacillus subtilis*, GabR activates the P_GabTD_ promoter in the presence of GABA (Nardella et al., 2020). Here, we report the first engineered biosensors for propionate and GABA in EcN. We show that the sensor prototyping constructs for each metabolite successfully identified the optimal promoter input and a final sensor design meeting the required sensitivity, dynamic range, and ON/OFF promoter output. The selectivity of both sensors was assayed, and no activation by non-cognate ligands was observed. The EcN GABA biosensor required the design of a synthetic GabR-regulated promoter, and we demonstrate the essentiality of the promoter elements for activation by GABA. The EcN propionate and GABA biosensors are high performance sensors that have 59-fold and 138-fold activation, respectively.

## Materials and Methods

### Strains, Media, and Inducers

*E. coli* Nissle 1917 was used for experimentally assaying sensors, and *E. coli* NEB 5-alpha (New England Biolabs) was used for cloning. EcN containing genetic sensors were assayed in M9 minimal media (Sigma-Aldrich; 6.78 g/L Na_2_HPO_4_, 3.0 g/L KH_2_PO_4_, 1.0 g/L NH_4_Cl, 0.5 g/L NaCl final concentration) with 0.34 g/L thiamine hydrochloride (Sigma-Aldrich), 0.2% casamino acids (Acros), 2 mM MgSO4 (Sigma-Aldrich), 0.1 mM CaCl_2_ (Sigma-Aldrich), and 0.4% D-glucose (Sigma-Aldrich). The antibiotic used to select for sensor plasmids was 50 mg/ml kanamycin (GoldBio). The various inducers used in this work were anhydrotetracycline hydrochloride (aTc; Sigma-Aldrich), gamma-aminobutyric acid (GABA; Sigma-Aldrich), sodium propionate (Sigma-Aldrich), acetic acid (Fisher Chemical), sodium butyrate (Sigma-Aldrich), 1-4 butanediol (Sigma-Aldrich), and L-glutamate (Sigma-Aldrich). Plasmids used in this work are listed in Supplementary Table S1. Genetic parts used in this work are listed in Supplementary Table S2, and sequences are provided in a synthetic biology open language (SBOL) format xml file (Supplementary File).

### Genetic Sensor DNA Assembly

Destination plasmids for each sensor were built using Type IIS DNA assemblies performed in two steps. First, a sensor cassette plasmid was made using the enzyme BbsI (New England Biolabs) by combining a promoter part, a ribozyme part, a ribosome binding site, the protein coding sequence for the regulator in the sensor, and a terminator. The sensor cassette was then combined with the PCR products of a backbone plasmid pLW555 (Andrews et al., 2018) containing *lacI*, *tetR*, and kanamycin resistance cassette (kanR) in a Type IIS DNA assembly reaction with SapI (New England Biolabs). To construct the sensor characterization plasmids, a sensor promoter and a standard yellow fluorescent protein (eYFP) output fragment from the pAN1717 standard plasmid (Nielsen et al., 2016) were assembled onto the sensor backbone plasmid in a BbsI Type IIS DNA assembly reaction. Orthogonal linker sequences for Type IIS assemblies were designed using a Python script (Woodruff et al., 2017).

Type IIS DNA assembly reactions were performed in 5 µl total volume containing 20 fmol of each purified DNA fragment (plasmid or PCR product or 25 ng of two annealed oligos), 10 fmol of the purified destination vector PCR product, 5 U of the appropriate Type IIS enzyme, and 0.25 µl T4 DNA ligase (20 U/µl; New England Biolabs) in 1X T4 DNA ligase buffer (New England Biolabs). The reaction mixture was incubated in a thermal cycler with the following protocol: alternating steps of 16°C for 5 min and 37°C for 5 min for 30 cycles, followed by 50°C for 30 min, and inactivated at 80°C for 10 min. Then, 2 µl of the assembly reaction was transformed into 5 µl of chemically competent cells (*E. coli* NEB 5-alpha, New England Biolabs).

The *gabR* and P_GabTD_ DNA fragments were PCR amplified from purified genomic DNA of the strain *Bacillus subtilis* 168. The *prpR* gene and P_PrpB_ promoter were amplified from the plasmid pPro24 (Lee and Keasling 2005) with a silent mutation incorporated to remove a BbsI recognition sequence.

### Sensor Characterization Assays

Genetically encoded sensors were transformed into electrocompetent *E. coli* Nissle 1917 using electroporation. To assay genetically encoded sensors, one colony was inoculated into 200 µl of M9 media with appropriate antibiotics in a U-bottom 96 well microtiter plate sealed with a breathable seal and incubated in a plate shaker at 37°C and 1,000 rpm for 16 h (Elmi DTS-4 microplate shaker). Two serial dilutions of 15 µl of culture into 185 µl of M9 minimal media with antibiotics were then performed and the cells were incubated for 3 h under identical conditions. Cells were then further diluted with two serial dilutions, the first one being 15 µl of cells into 185 µl of M9 minimal media with antibiotics and the second being 3 µl of the diluted culture into 145 µl of M9 minimal media with the appropriate antibiotics and the appropriate inducer concentration added to the medium. After 5 h of incubation, the cell fluorescence of each sample was measured via flow cytometry. To measure cell fluorescence, an aliquot of cells was diluted into phosphate buffered saline with 2 mg/ml kanamycin and incubated at room temperature for 30 min before flow cytometry analysis. The concentrations of propionate used for the sensor characterization curves were 40 mM, 30 mM and then a 2-fold dilution factor for each subsequent concentration. The GABA concentration used was 50 mM and decreased using a 2-fold dilution factor for each subsequent concentration.

### Flow Cytometry Analysis

Fluorescence was measured using a BD Accuri C6 flow cytometer using the 20 mW 480 nm solid state blue laser. The data for each sample was collected with a cutoff of 10,000 gated cell events with at least 5,000 events collected per sample at a flow rate of less than 1,000 events/s for all samples. The cells were gated with a gate for cell-sized particles using the FlowJo software. The geometric median cell fluorescence was calculated in FlowJo.

Arbitrary units of cell fluorescence were converted to standard relative promoter units (RPU) as previously described (Nielsen et al., 2016; Andrews et al., 2018). The conversion to RPU is as follows:

$$RPU=\left(YFP\;-\;YFP_{o}\right)/\left(YFP_{RPU}\;-\;YFP_{o}\right)\;$$ (1)

where YFP is the median fluorescence of the sample, YFP_o_ is the median fluorescence of wildtype EcN cells lacking a plasmid, and YFP_RPU_ is the median fluorescence of EcN cells harboring the standard plasmid pAN1717, which contains *eYFP* expressed by a constitutive promoter. Samples with fluorescence equal to or below the autofluorescence of wildtype EcN cells were plotted at a value of 0.001 RPU, set to be the limit of detection of our assay.

To determine the response function for the inducible P_Tet_ promoter, the measured sensor output in RPU was fit to the Hill equation Eq. 2 (Ang et al., 2013). The fit was performed using the least squares method, minimizing the sum of the log_10_ of the error magnitudes between the curve fit and the data points. This was done using the solver add-in in Microsoft Excel with the GRG nonlinear solving method.

$$y=y_{min}+\frac{\left(y_{max}-y_{min}\right)x^{n}}{k^{n}+x^{n}}$$ (2)

## Results

### Approach for Rapid Prototyping of Metabolite Biosensors

**FIGURE 1:**
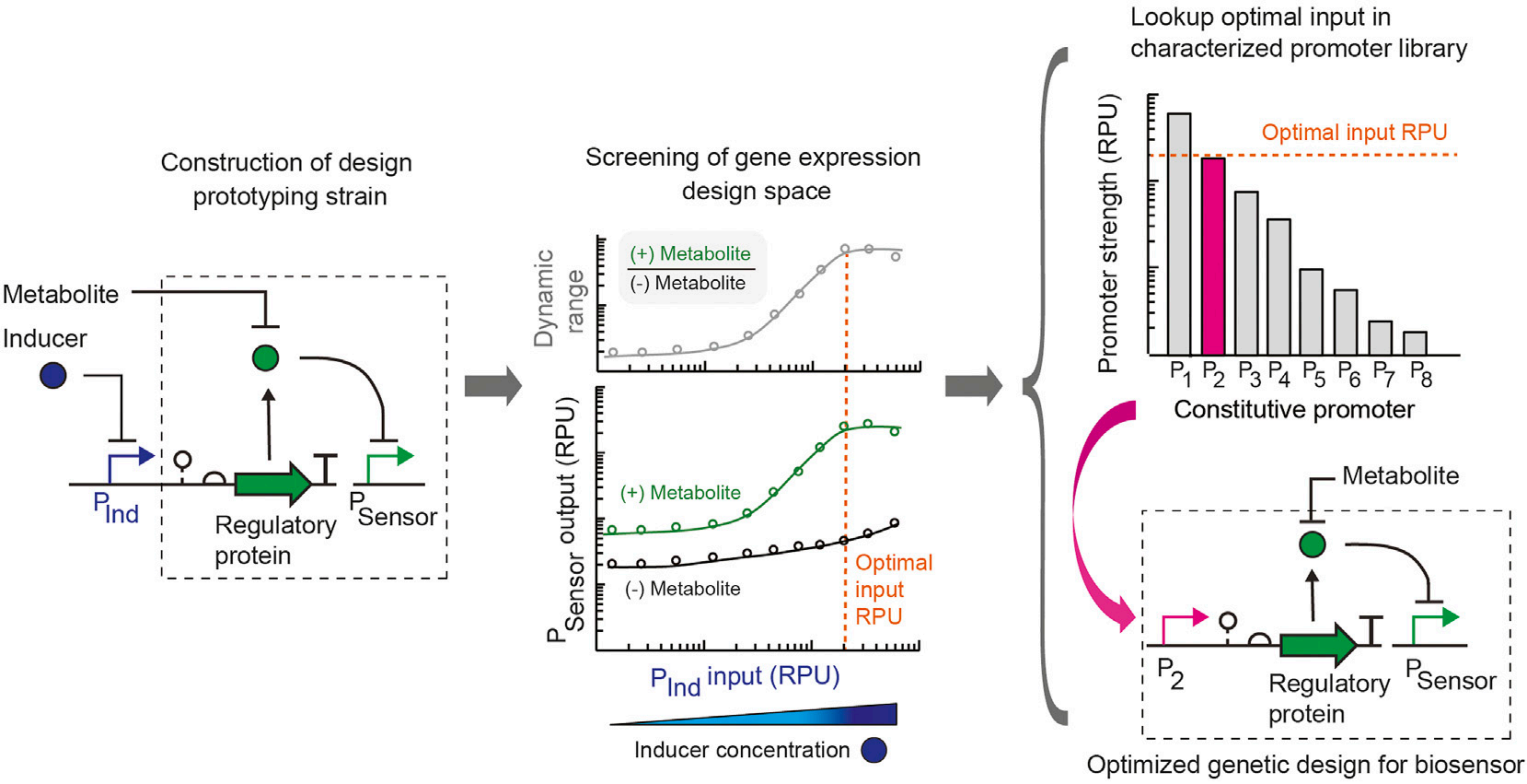
Rapid design prototyping approach for engineering metabolite biosensors. Initially, a design prototyping construct is built for the whole cell biosensor, which uses a tightly-regulated and characterized inducible promoter (P_Ind_) to control the expression of the metabolite-responsive transcription regulatory protein. A ribozyme part is used to genetically insulate the design. The sensor output promoter (P_Sensor_) is specified, and the regulator for the P_Ind_ promoter is expressed on the construct. The target for the sensor’s sensitivity is selected based on the metabolite concentration to be detected in the application of interest. For use in the human gut, the metabolite concentration to be detected in the gut is specified. Then, the sensor prototyping design is assayed in parallel experiments for the range of transcription regulator expression levels for each condition with addition of the target metabolite concentration (green) and without the addition of the metabolite (black). The sensor output is measured in standard relative promoter units (RPU). From this dataset, an optimal promoter input value (orange dotted line) is selected that achieves the required sensitivity, optimal dynamic range (ratio of ON promoter output /OFF promoter output) and suitable promoter output in the activated and unactivated states. From a library of constitutive promoters characterized in standard units (RPU), a promoter part that matches the optimal input value (orange dotted line) is selected from a library and used to replace the inducible promoter to build the final engineered sensor design. The final design is optimized for the specified sensitivity of the sensor.

We introduce a simple approach to simultaneously assay and optimize multiple attributes of a metabolite biosensor’s performance. Whereas biosensor engineering often employs trial-and-error and the construction of large libraries of design variants, we present the use of a design prototyping strain to assay a range of gene expression levels in parallel for each sensor promoter by expressing the metabolite-responsive transcription factor using an inducible promoter with a large dynamic range. In combination with an insulated genetic architecture and strain-specific library of promoters characterized using a standard unit, a single final sensor design can be built by substituting the constitutive promoter part that achieves the optimal input promoter activity (Figure 1). In this way, the DNA construction required is minimal. For a transcription factor-based metabolite biosensor, key attributes of the sensor that determine its suitability for an application are its: *(i)* sensitivity (metabolite concentration that activates the sensor), *(ii)* dynamic range (ratio of the sensor output promoter activity in the activated ON and unactivated OFF states), *(iii)* basal activity or leakiness (sensor output promoter activity in the OFF state), and *(iv)* selectivity for the metabolite (absence of cross-reactivity with other ligands). The expression level of the metabolite-responsive transcription factor plays a key role in determining the sensitivity and dynamic range of the sensor in addition to its basal activity. Another consideration in some cases is that the gene expression level of the transcriptional regulator can introduce toxicity and inhibit growth if expressed too highly (Ding N. et al., 2021).

Here we aimed to rapidly probe the gene expression design space and its relationship to these attributes for metabolite biosensors using this strategy in the probiotic bacterium *E. coli* Nissle 1917. Importantly, for a metabolite biosensor to be used as a living diagnostic in the gut, the properties and sensitivity of the sensor must match the *in vivo* metabolite concentrations that will be detected. Common sensor engineering strategies that alter the ligand concentration to achieve the optimal sensor performance are not adequate for these applications. Instead, here we specified the required sensor sensitivity at the outset based on the *in vivo* metabolite concentration to be detected. Using a design prototyping strain for the sensor, we identified an optimal promoter input for the metabolite-responsive transcription factor that achieves a sufficiently large dynamic range at the target metabolite concentration. The final sensor design was then created by selecting a constitutive promoter from a library of characterized promoters that corresponds to the optimal input and using this promoter part to replace the inducible promoter in the design prototyping construct.

For the sensor prototyping construct, we selected the P_Tet_ inducible promoter, which has a very low basal expression and large dynamic range of 479-fold activation (Supplementary Figure S1). This allowed us to scan a wide range of expression levels to find one that provides an appropriate dynamic range and output level for the sensor while only requiring the construction of two sensor variants, one using the inducible promoter to express the transcription factor and the final sensor construct. Importantly, the genetic design for this approach includes a self-cleaving ribozyme as a genetic insulator downstream of the input promoter that expresses the regulatory protein component of the sensor. Central to our approach is the substitution of a tightly-regulated inducible promoter (used for prototyping) to a constitutive promoter in the final sensor design based on the promoter characterization of all promoters in a standard relative promoter unit (Nielsen et al., 2016; Andrews et al., 2018). Therefore, an insulated genetic design with a self-cleaving ribozyme (e.g., RiboJ) is critical to have an identical 5’ untranslated region of the transcript containing the transcription factor and maintain the relative promoter strength when substituting promoter parts (Lou et al., 2012; Nielsen et al., 2016).

### A Synthetic Optimized Propionate Sensor for EcN

**FIGURE 2:**
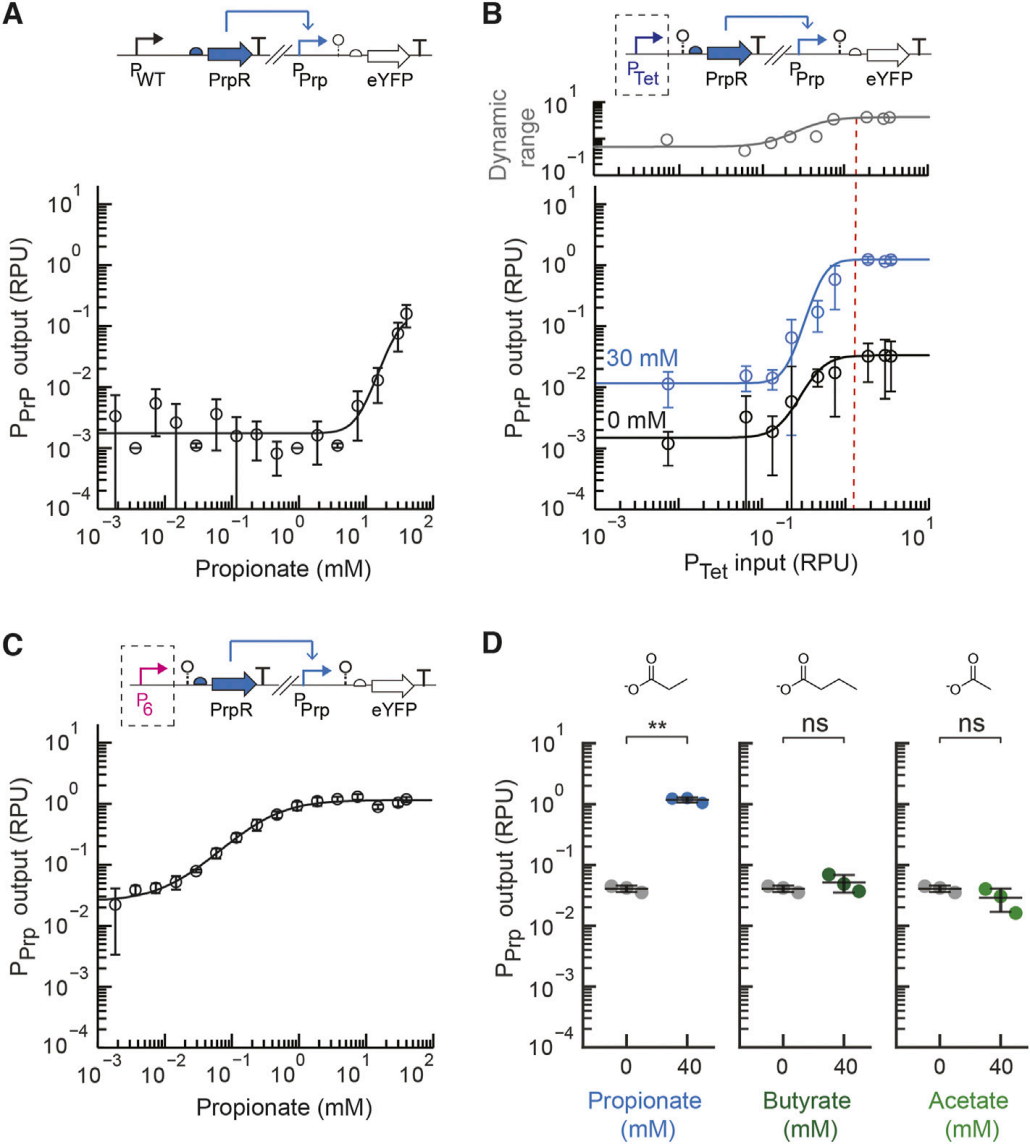
Engineering a propionate sensor for EcN. **(A)** The measured sensor response of the initial propionate sensor design (pML 2004) containing the native *E. coli* promoter expressing PrpR (P_WT_). The experimental measurements were fit to the Hill equation. **(B)** The prototyping design for the propionate sensor with inducible expression of PrpR by P_Tet_ (pML2002) was assayed for a range of inducer concentrations (aTc) with 30 mM of propionate added (blue) or no propionate added (black). The plotted dynamic range was calculated as the ratio of the average promoter output with propionate to without propionate. The dotted line represents the optimal promoter input region selected. **(C)** The measured sensor response of the final propionate sensor (pML2003) containing the constitutive P_6_ promoter to express PrpR. **(D)** Selectivity of the final propionate sensor design was assayed by measuring the sensor output without (grey) and with exogenous addition of propionate, butyrate, or acetate (40 mM) to the medium. For all panels, the cell fluorescence was measured via flow cytometry, and arbitrary fluorescence units were converted to standard RPU (Methods). The bars and markers represent the average of the measured geometric median of a population of at least 5,000 cells assayed in three identical experiments performed on three different days. All error bars represent the standard deviation. Student’s *t*-tests (paired, two-tailed) were performed, and *p*-values ≤ 0.01 (**) and ≤0.05 (*) are indicated.

We first aimed to develop a propionate metabolite sensor for EcN that could detect the amount of propionate generally found in the intestines, which ranges from approximately 5–30 mM (Cummings et al., 1987; den Besten et al., 2013). We started by utilizing the *E. coli* PrpR protein sequence and PrpR-regulated P_PrpB_ promoter sequence, selected to be the cognate P_Prp_ promoter, from the previously developed pPro24 plasmid (Lee and Keasling 2005). In this propionate biosensor strategy developed by the Keasling lab, the endogenous expression of PrpEC converts propionate into 2-methylcitrate, and 2-methylcitrate activates the PrpR transcriptional regulator. An initial propionate sensor construct, pML2004 (Supplementary Figure S2), was constructed that used these DNA parts to construct a sensor construct on a low copy plasmid (p15a origin of replication) with the native *E. coli* promoter expressing PrpR (P_WT_) and the native RBS for PrpR. In their native genetic context and prior *E. coli* propionate sensor, the promoter expressing PrpR and the PrpR-regulated output promoter are overlapping divergent promoters. We chose to separate these promoters and place them at distant locations on the plasmid to prevent possible transcriptional interference and facilitate their future use. The sensor output promoter activity was measured in standard relative promoter units by placing an insulated standard yellow fluorescent protein (eYFP) fragment downstream of the sensor output promoter (Nielsen et al., 2016; Andrews et al., 2018). This initial sensor construct displayed a low output promoter activity even with high concentration of propionate (40 mM) and negligible activation with the addition of 3.75 mM propionate (Figure 2A). Increasing the propionate concentration beyond 40 mM significantly inhibited cell growth of EcN.

Next, the design prototyping construct for the propionate sensor was built (plasmid pML2002) in which the PrpR transcriptional regulator was expressed by the inducible P_Tet_ promoter. The native RBS for PrpR was again used. We assayed the range of expression levels for PrpR by increasing the induction levels of P_Tet_ with anhydrotetrycycline (aTc) for conditions without and with the addition of the target propionate concentration (30 mM). For all sensor characterization assays, previously developed standard characterization protocols were used (Nielsen et al., 2016; Andrews et al., 2018). From the prototyping assay, the input promoter activity that achieved a high activated sensor output and large dynamic range under the specified conditions was identified (Figure 2B). A promoter input of approximately 1.3 RPU showed the greatest dynamic range, while higher input promoter activity showed no significant change in the dynamic range or output. Six constitutive promoters were characterized in EcN to form a small library (Supplementary Figure S1). These promoters are based on the J23119 Anderson promoter with synthetic sequences that were introduced downstream of −35 region to alter its promoter strength (Supplementary Table S2).

From this promoter library, the P_6_ promoter part (4.0 ± 0.4 RPU) was selected to replace P_Tet_ and create the final propionate sensor design (pML2003 plasmid). In both the prototyping and final sensor designs (pML2002 and pML 2003, respectively) the ribozyme RiboJ53 was placed immediately downstream of the P_Tet_ or P_6_ promoter, respectively, to insulate the genetic design (Lou et al., 2012; Nielsen et al., 2016). The sensor response function for the final EcN propionate sensor design was assayed as above using a standard 5-hour induction time (Figure 2C). With 30 mM propionate, the dynamic range of the sensor is 59-fold activation, while the basal activity is very low at 0.04 ± 0.005 RPU. Moreover, the sensor achieved its fully activated output of 1.3 ± 0.1 RPU with only 7.5 mM propionate, demonstrating it was engineered to have the necessary sensitivity. For comparison, the initial design with P_WT_ produced only 1.5-fold activation and a very low sensor output (0.005 RPU) when induced with this concentration of propionate.

Importantly, glucose catabolite repression has been reported for the endogenous PrpR promoter and P_PrpB_ promoter, which are activated by cAMP receptor protein (CRP) (Lee et al., 2005; Lee and Keasling 2005). Here we used a synthetic promoter to express to PrpR to alleviate endogenous catabolite repression. The P_Prp_ sensor output promoter contains a CRP binding site overlapping the PrpR operator, which was not removed because prior work showed that mutation abolished regulation by PrpR (Lee et al., 2005). While the absence of glucose is expected to increase basal expression of the sensor and reduce the dynamic range, the propionate sensor is expected to maintain a reasonably high dynamic range in the presence 2–50 mM glucose, which is typically reported for luminal intestinal concentration in animals and humans (Olsen and Ingelfinger 1968; Ferraris et al., 1990).

To test whether the propionate sensor is suitable for use in the presence of other metabolites found in the human gut, we next assayed the selectivity of our engineered propionate sensor. The activation to acetate and butyrate were assayed since these two short chain fatty acids share a similar chemical structure and are present in significant amounts in the intestines (Parada Venegas et al., 2019). The engineered propionate sensor was highly selective for propionate showing no significant induction in the presence of 40 mM acetate or 40 mM butyrate (Figure 2D). This is consistent with the fact that propionate indirectly induces P_prp_ via the 2-methylcitrate-responsive PrpR regulator (Palacios and Escalante-Semerena 2004), and this indirect activation may be beneficial for the selectivity. Given that the concentration of acetate and butyrate in the human gut has been reported to be less than 40 mM (Parada Venegas et al., 2019), this suggests that the selectivity of the engineered propionate sensor is sufficient.

### A Synthetic Optimized GABA Sensor for EcN

Next, we aimed to develop a biosensor for GABA in EcN using our design prototyping approach. Notably, a whole cell biosensor for GABA has not been reported in prior work to date in EcN or another Gram-negative bacterium. We utilized the GabR transcriptional repressor from *B. subtilis*, which is a member of the MocR subfamily of GntR transcription regulators that responds to increasing GABA concentration by upregulating the biosynthesis of glutamate from GABA in *B. subtilis*. In the absence of GABA, GabR is an autorepressor (Belitsky and Sonenshein 2002; Edayathumangalam et al., 2013). GabR is an interesting transcriptional regulator that contains an aminotransferase domain and has been shown to bind both pyridoxal-5′-phosphate (PLP) and GABA, which react to form an aldimine that activates GabR-mediated transcription of the P_GabTD_ promoter *via* a dimeric GabR protein complex (Edayathumangalam et al., 2013; Okuda et al., 2015; Wu et al., 2017). The P_GabTD_ promoter has been well-studied and was proposed to have three putative DNA binding sites for GabR, comprised of two 6-nt direct repeats that have an inverted repeat between them (Edayathumangalam et al., 2013; Nardella et al., 2020). This 41 bp region that has been suggested to bind GabR overlaps the divergent promoters for *gabTD* and *gabR* on the *B. subtilis* genome.

To design a synthetic GabR-regulated promoter for EcN that maintained the three putative GabR binding sites and their native spacing in P_GabTD_, a synthetic promoter was designed that integrated the three putative binding sites and an *E. coli* constitutive promoter. The Anderson promoters J23105 and J23119 were selected as the *E. coli* core promoter parts (Kelly et al., 2009) to generate the synthetic P_Gab105_ and P_Gab119_ promoters parts. In these promoters, the −35 sequence of the core *E. coli* promoter was placed between the second and third putative GabR binding sites (S2 and S3), and the sequence between the −10 and −35 regions was changed to incorporate the third binding site (S3). The native upstream element (UE) sequence between the first and second putative GabR binding sites (S1 and S2, respectively) was included in the synthetic P_Gab_ promoter design for EcN. This general strategy of combining elements to form a new synthetic sensor output promoter for *E. coli* has been employed previously (Siedler et al., 2014; Liu et al., 2020).

**FIGURE 3:**
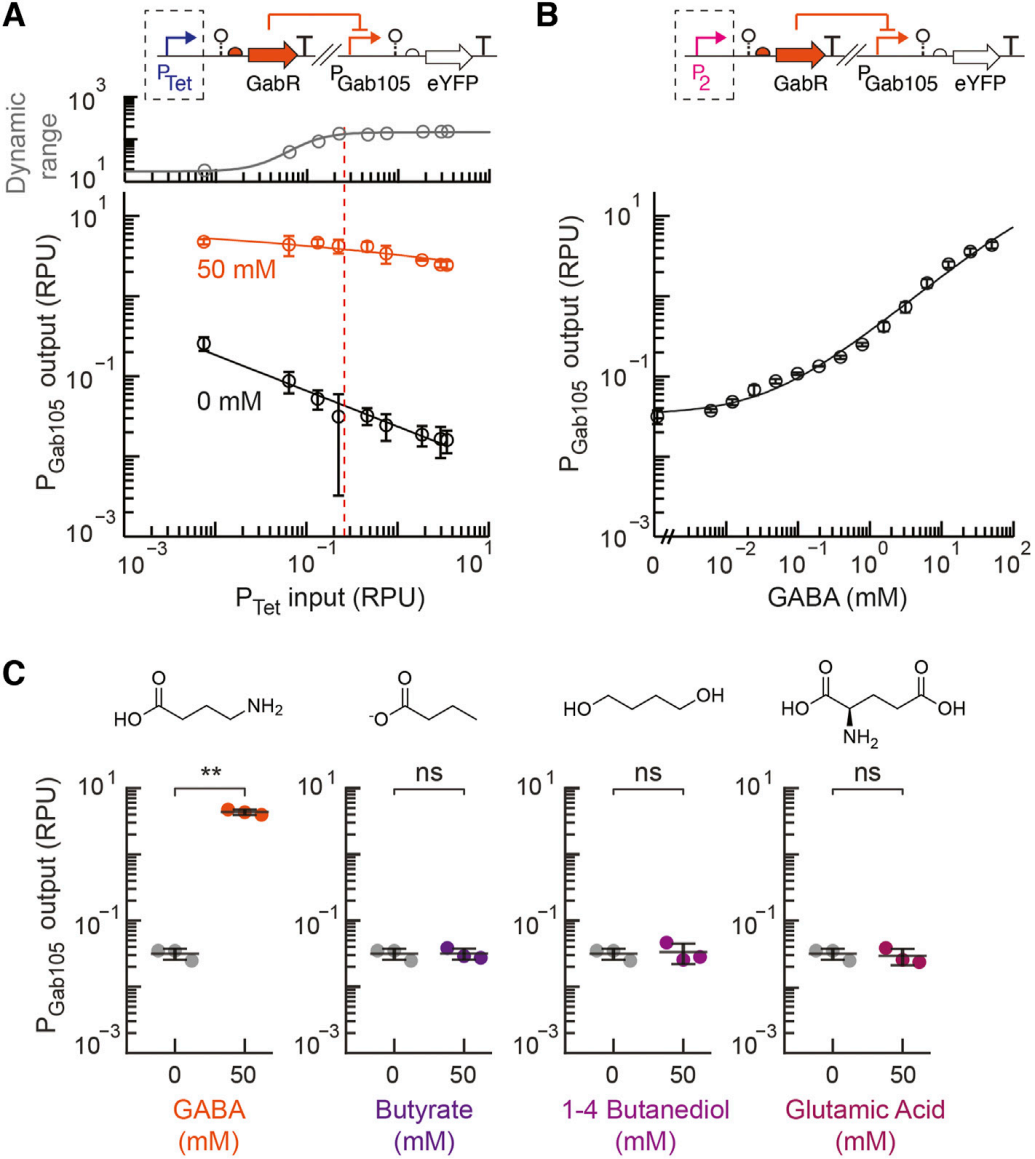
Engineering a GABA sensor for EcN. **(A)** The prototyping design for the GABA sensor (pML3002), which contains the synthetic GabR-regulated P_Gab105_ promoter and inducible GabR by P_Tet_, was assayed for a range of inducer concentrations (aTc) with 50 mM GABA exogenously added (blue) to the medium or without GABA addition (black). The plotted dynamic range was calculated as the ratio of the average promoter output with GABA addition to without GABA. The dotted line shows the selected optimal promoter input for the selection of the constitutive P_2_ promoter. **(B)** The response of the final GABA sensor design (pML3009) to GABA was assayed and fit to the Hill equation. **(C)** The selectivity of the final GABA sensor design (pML3009) was assayed with exogenous addition of 50 mM of either GABA, butyrate, 1–4 butanediol, or glutamic acid and compared to the medium without metabolite addition. For all panels, the cell fluorescence was measured *via* flow cytometry, and arbitrary fluorescence units were converted to standard RPU (Methods). The bars and markers represent the average of the measured geometric median of a population of at least 5,000 cells assayed in three identical experiments performed on three different days. All error bars represent the standard deviation. Student’s *t*-tests (paired, two-tailed) were performed, and *p*-values ≤ 0.01 (**) and ≤0.05 (*) are indicated.

Next, the prototyping design construct for the GABA sensor was designed with the inducible P_Tet_ promoter used to control the expression of the insulated *gabR* sequence with a synthetically designed ribosome binding site (Reis and Salis 2020; Cetnar and Salis 2021) for each synthetic P_Gab_ promoter (P_Gab105_ and P_Gab119_ on plasmids pML3002 and pML3003, respectively). There are limited studies that report the concentration of GABA in the human intestines. However, a recent study reported a fecal concentration of 0–300 mM, which was estimated using a previously reported conversion factor for the density of fecal matter, with the majority of participants between 0 and 100 mM (Brown et al., 1996; Altaib et al., 2021). Based on these reports, we selected 50 mM GABA as our target sensitivity for the sensor. Each GABA prototyping strain was assayed in parallel experiments to measure the sensor output with and without GABA addition (50 mM) over a 479-fold range of induction for P_Tet_ regulating the expression of GabR. For the P_Gab119_ prototyping construct, a high basal output and small dynamic range were observed over the entire range of induced GabR expression (Supplementary Figure S3). The prototyping sensor design containing the synthetic P_Gab105_ promoter showed a higher ON state output and much lower basal activity that contributed to a large dynamic over the range of P_Tet_ induction (Figure 3A). As the P_Tet_ input increased up to 0.2 RPU, the sensor’s dynamic range increased and approached its maximum dynamic range. Increasing the GabR expression beyond this point had the undesirable effect of decreasing the ON state output.

For the final engineered GABA sensor design, we chose the P_2_ constitutive promoter, which has a strength of about 0.2 ± 0.02 RPU, to express GabR. While an even larger dynamic range was observed for greater promoter input values, we also observed greater cell-to-cell variability in the activated ON state of the sensor at higher GabR expression levels, as observed by some bimodality in the single-cell fluorescence distribution measured by flow cytometry (Supplementary Figure S4). Therefore, we chose a moderate input promoter activity that resulted in a sensor design with a sufficiently high dynamic range and low basal activity. The sensor response function for this final GABA sensor design (pML3009) was measured and showed a very large dynamic range of 138-fold and a high ON state output promoter activity of 4.3 ± 0.4 RPU (Figure 3B). For comparison, the native GabR system for sensing GABA in *B. subtilis* was reported to produce approximately 5-fold activation over 4 h (Nardella et al., 2020). We also tested the activity of the synthetic P_Gab_ promoters (P_Gab105_ and P_Gab119_) on a backbone that did not contain *gabR* (pML3010 and pML3011, respectively). We observed both promoters having high output in the absence of GabR expression that is equivalent to the fully activated GABA sensor output (Supplementary Figure S5).

We assayed the selectivity of our engineered GABA biosensor for GABA relative to other possible inducers that have similar chemical structures and might be found in the same environment that the sensor would be used in. Glutamate and butyrate were chosen since both can be found in the intestinal tract, while 1-4 butanediol was examined as it is a precursor in the synthesis of other chemicals. Our engineered GABA biosensor containing the synthetic P_Gab105_ promoter showed a high specificity for GABA with no activation detected for the other metabolites when added at a high concentration of 50 mM (Figure 3C).

**FIGURE 4:**
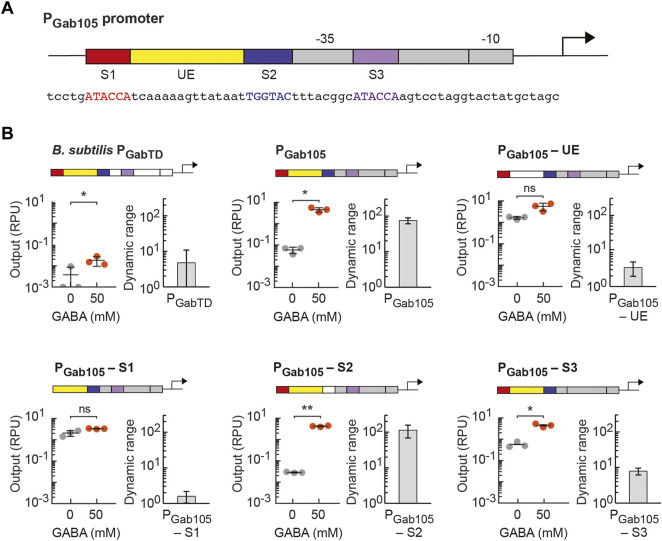
Identifying essentiality of promoter elements in synthetic P_Gab_ promoters regulated by GabR. **(A)** Genetic schematic of the synthetic P_Gab105_ promoter designed for *E. coli*, which contains three putative GabR binding sites (S1, S2 and S3, respectively) and the upstream element (UE) from the native GabR-regulated P_GabTD_ promoter in *B. subtilis*. The *E. coli* core promoter sequence (grey) is based on a constitutive promoter (J23105 from the Anderson collection). The sequence between the –10 and –35 regions was designed to contain binding site S3 (purple), while the binding sites S1 (red) and S2 (blue) and the UE (yellow) were placed upstream in the promoter sequence. The promoter sequence is also shown. **(B)** Variants of the P_Gab105_ promoter were designed to remove either one of the putative binding sites (S1, S2, or S3) or the UE. Each of the promoter variants or the native *B. subtilis* P_GabTD_ promoter were assembled into the pML3000 prototyping backbone, which contains GabR under the control of P_Tet_. The resulting six GABA sensor constructs, each containing a different P_Gab_ promoter, were assayed with 0.125 ng/ml aTc (0.064 RPU) to induce GabR expression with either 50 mM GABA addition (orange) or without addition (grey) to the medium. The cell fluorescence was measured via flow cytometry, and arbitrary fluorescence units were converted to standard RPU (Methods). The circles represent measured geometric median of a population of at least 5,000 cells assayed in identical experiments performed on three separate days with the bar indicating the average of the replicates. The dynamic range for the experiment on each day was calculated as the ratio of the ON to OFF sensor output, and the error bars represent the standard deviation. Student’s *t*-tests (paired, two-tailed) were performed, and *p*-values ≤ 0.01 (**) and ≤0.05 (*) are indicated.

Given the high activity of our synthetic GABA biosensor in EcN, we next sought to determine which elements of the synthetic P_Gab_ promoter design are necessary for the activity of the GabR-mediated GABA sensor. Starting with our P_Gab105_ synthetic promoter design (Figure 4A), we created variants of this promoter in which we individually removed one of the three putative GabR binding sites (S1, S2, S3) or the upstream element (UE) from the promoter’s DNA sequence (promoter sequences listed in Supplementary Table S2). For characterization of each sensor design, the P_Gab_ promoter variant was assembled into the pML3000 backbone containing GabR under the control of P_Tet_ and assayed with GabR expression induced with 0.125 ng/ml aTc (0.064 RPU). To start, we tested the activity of a sensor design containing the wildtype *B. subtilis* P_GabTD_ promoter in EcN and observed very low activation upon GABA addition (Figure 4B). In the P_Gab105_-UE promoter variant, the 15 bp UE sequence was substituted with a randomly generated 15-bp sequence, and this sensor displayed loss of GABA activation, indicating UE is a necessary element for the sensor (Figure 4B). This finding is consistent with the reported importance of the flexible DNA between the two direct repeat GabR binding sites that allows for shape recognition and binding in the GabR-DNA interaction (Al-Zyoud et al., 2016). The removal of the first direct repeat GabR binding site (S1) in the P_Gab105_-S1eliminated GABA activation (Figure 4B). In the P_Gab105_-S2 variant, the 6-bp indirect repeat putative binding site (S2) was mutated from TGGTAC to ACCTAC, and interestingly, this did not reduce GABA activation of the sensor (Figure 4B). Lastly, we removed the S3 direct repeat binding site and replaced it to integrate the full J23105 sequence in the P_Gab105_-S3 promoter variant, and this sensor showed reduced but still significant activation (Figure 4B). Overall, these finding are generally consistent with reports that the direct repeat binding sites (S1 and S3) participate in GabR binding while the shape and flexibility of DNA between these sites facilitates GabR binding and regulation (Amidani et al., 2017). However, it is notable that we observe the GABA sensor maintains some functionality even when promoter elements previously reported to be necessary for GabR-mediated regulation are mutated.

## Discussion

The widespread realization of living bacterial strategies to detect and regulate metabolites in the gut requires efficient approaches to engineer highly functional metabolite biosensors for gut bacteria that sense physiologically relevant metabolite concentrations. Using a rapid prototyping approach for transcription factor-based biosensors, we demonstrated that propionate and GABA sensors can be engineered and optimized for EcN with little DNA construction or permutation of genetic parts required. We showed that by using well-insulated genetic designs and well-characterized promoter parts, we can utilize a design prototyping construct and inducible expression of the metabolite-responsive regulator to assay the sensor response across the full range of gene expression and identify the optimal promoter input strength in standard units. The final biosensor design is then created by selecting a constitutive promoter of this strength to replace the inducible expression of the transcriptional regulator. Importantly, the necessary sensitivity of the biosensor is specified at the outset, and the experimental assaying of the gene expression design space is performed at these relevant *in vivo* concentrations. We demonstrated that this sensor engineering approach is also useful when a synthetic output promoter, which is regulated by the sensor’s allosteric transcription factor, must be engineered for the organism.

We report what we believe are the first optimized sensors for propionate and GABA in EcN. For the engineered EcN propionate sensor, tuning the expression of PrpR was able to increase the sensitivity of the sensor >500-fold, such that the sensor is activated by typical *in vivo* concentrations of 5–30 mM propionate in the human gut. These improvements in the sensitivity and activated sensor output are especially important to integrate the propionate sensor within engineered regulatory networks and utilize existing components for genetic circuit design. The *in vivo* behavior of the sensor presents many questions, as changes in growth, oxygen, and metabolism may affect sensor performance, and are generally difficult to predict. The general framework for sensor engineering that we present here may be a useful strategy for multi-factorial optimization of sensor activity in a range of environmental conditions, such as the variable conditions within the gastrointestinal tract, using a minimal number of DNA constructs.

Next, we demonstrated that a synthetic GABA biosensor could be engineered in EcN using the GabR regulator from *B. subtilis* and previously identified putative binding sites for GabR. Experimentally surveying the expression space for GabR using two GABA sensor prototyping constructs (containing two synthetic promoter designs) identified a suitable design that achieves high activation at typical *in vivo* concentrations (50 mM GABA). The EcN GABA biosensor has a large 138-fold dynamic range. Further experiments helped to identify the required elements of the synthetic GabR-regulated promoter. The selectivity of the propionate and GABA sensors was assayed, and both were found to be highly selective. Looking ahead, these metabolite sensors could be integrated into genetically-encoded circuits to create living therapeutics or diagnostics to interrogate their role in mood disorders and the gut-brain axis (Evrensel and Ceylan 2015; Mayer et al., 2015; Dinan and Cryan 2017; Bonaz et al., 2018; Cryan et al., 2019), while this sensor engineering strategy may be applied to a wide range of transcription factor-based biosensors and conditions that would enable *in situ* detection and programmable responses by living cells.

## Data Availability

The datasets presented in this study can be found in online repositories. The names of the repository/repositories and accession number(s) can be found in the article/Supplementary Material.
