# Supplementary material for: Surveying the Genetic Design Space for Transcription Factor-Based Metabolite Biosensors: Synthetic Gamma-Aminobutyric Acid and Propionate Biosensors in E. coli Nissle 1917
Source: Front Bioeng Biotechnol. 2022 Aug 25;10:938056. doi: 10.3389/fbioe.2022.938056 (PMC9452892; doi:10.3389/fbioe.2022.938056)
Supplement: Supplementary file 1 [file DataSheet1.pdf]

Contents

Supplementary Figure 1. Promoter characterization in *E. coli* Nissle 1917 2
Supplementary Figure 2. Plasmid maps for plasmids used in this work 3
Supplementary Figure 3. Sensor design prototyping for the P_{Gab119} promoter 4
Supplementary Figure 4. Cell fluorescence distributions for GABA sensor prototyping plasmid 5
Supplementary Figure 5. Synthetic P_{Gab} promoter output in the absence of *gabR* 6
Supplementary Table 1. Plasmid constructs used in this paper 7
Supplementary Table 2. Genetic part sequences used in this work 9
References 12

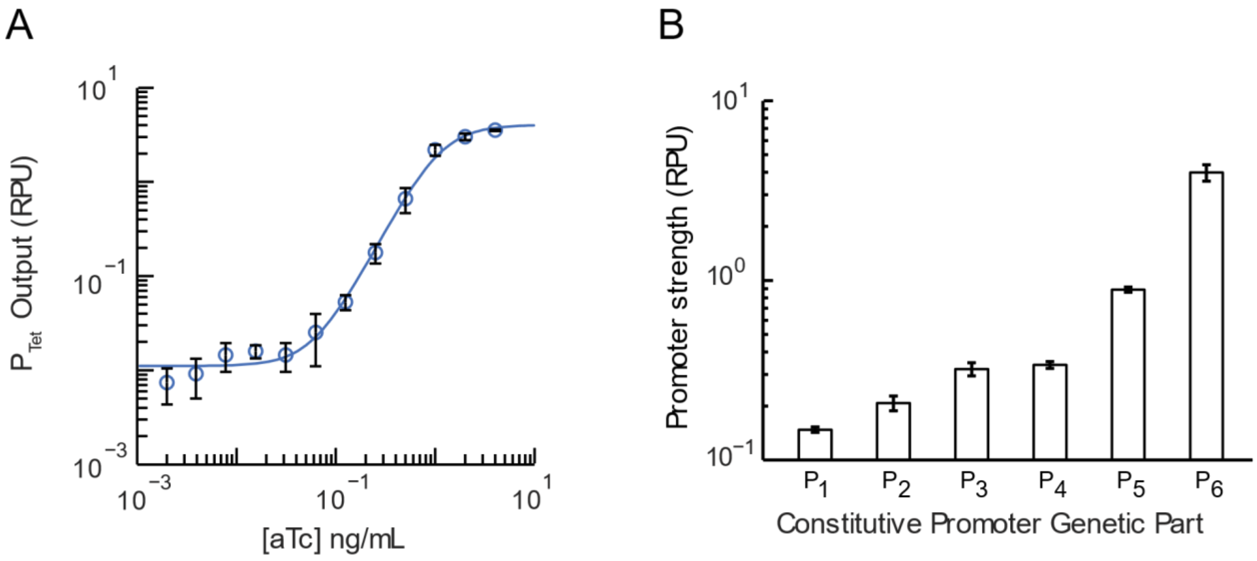

Supplementary Figure 1. Promoter characterization in *E. coli* Nissle 1917. **(A)** P_{Tet} sensor characterization was measured in *E. coli* Nissle 1917. Cells containing the sensor characterization plasmid (pT_pLW555[1], Supplementary Figure 2B) were assayed with a range of inducer (aTc) concentrations. Cell fluorescence was measured by flow cytometry. Promoter output for the P_{Tet} promoter was converted to standard relative promoter units (RPU) using standardized methods and the pAN1717[1,2] plasmid. **(B)** Constitutive promoter characterization was performed in *E. coli* Nissle 1917. Cells containing the promoter characterization plasmid (pML100X, Supplementary Figure 2A) for each constitutive promoter expressing a standard, insulated eYFP cassette were assayed. Cell fluorescence was measured via flow cytometry and converted to RPU units. Bars and markers represent the average of the measured geometric median for a population of 5,000-10,000 cells measured in three identical experiments performed on different days. Error bars represent the standard deviation (n = 3).

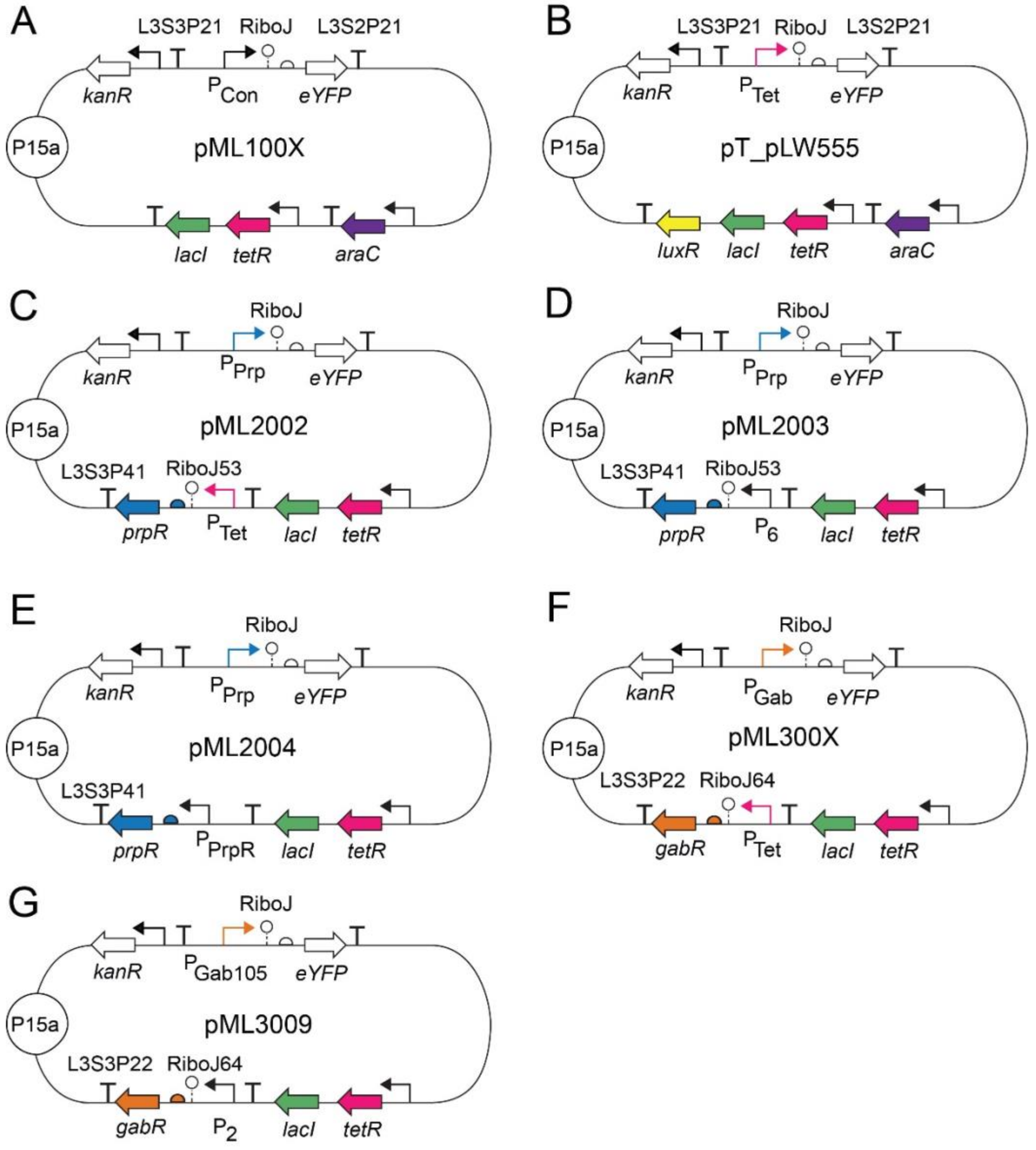

Supplementary Figure 2. Plasmid maps for plasmids used in this work. **(A)** Plasmid used to characterize each constitutive promoter (P_{Con}) in the library. **(B)** Sensor characterization plasmid for the P_{Tet} inducible promoter, which was used to determine output RPU[1] as a function of the inducer (aTc) concentration. **(C)** Design prototyping plasmid for the propionate sensor. **(D)** Final optimized propionate sensor plasmid construct. The selected constitutive promoter (P6) was integrated to express PrpR. **(E)** Design prototyping plasmid for the gamma-aminobutyric acid (GABA) sensor. **(F)** Final optimized propionate sensor plasmid construct. The selected constitutive promoter (P2) was integrated to express GabR.

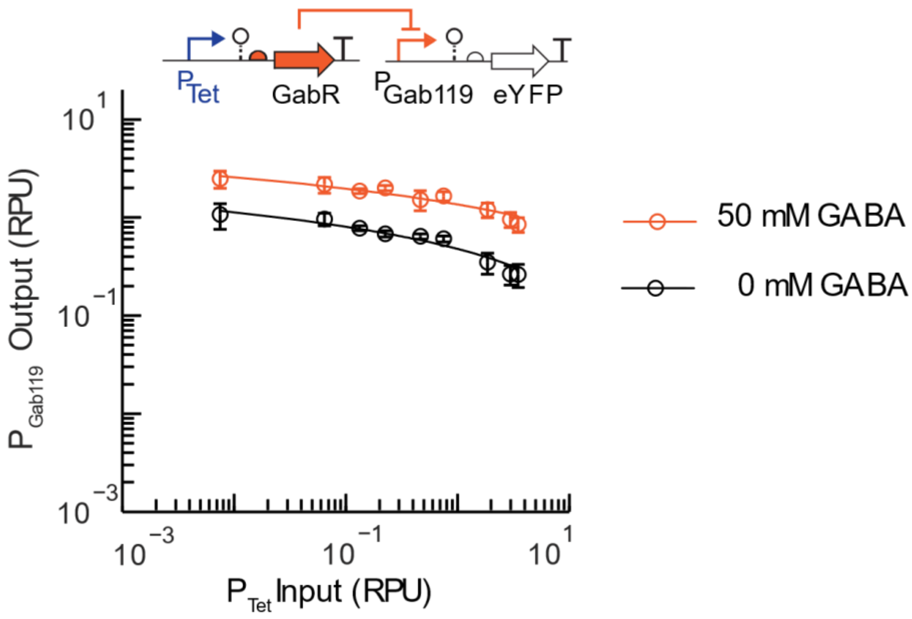

Supplementary Figure 3. Sensor design prototyping for the P_{Gab119} promoter. *E. coli* Nissle 1917 cells containing the GABA sensor design with the P_{Gab119} promoter (pML3003) were assayed using the GABA sensor characterization protocol. Samples were assayed with GABA addition to the medium (50 mM GABA, orange) and without GABA addition (black) and different inducer concentrations (aTc) to vary the expression of GabR. The promoter P_{Gab119} is a GABA sensing promoter variant that uses J23119 as the core *E.coli* promoter part, instead of the J23105[3] promoter used in the P_{Gab105} design. Cell fluorescence of each sample was measured via flow cytometry, and the sensor promoter output was converted to RPU units. The markers represent the average of the measured geometric median for a population of 5,000-10,000 cells each assayed in an identical experiment on three separate days. Error bars represent the standard deviation (n = 3).

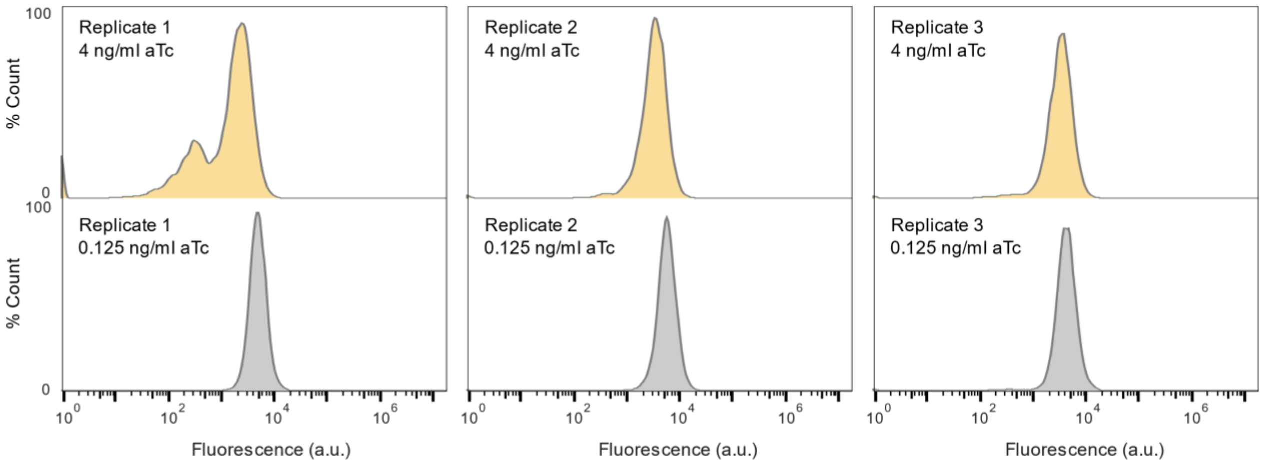

Supplementary Figure 4. Cell fluorescence distributions for GABA sensor prototyping plasmid. GabR, under the control of P_{Tet}, was induced with high expression 4 ng/ml aTc (top) or moderate expression 0.25 ng/ml aTc (bottom) in media containing 50 mM GABA. Three representative replicates from experiments are three separate days are shown. Cell fluorescence for a population of 5,000-10,000 cells was measured via flow cytometry. These distributions were used for Figure 3 to determine the geometric median for each sample and convert to RPU units. Cell counts are shown as the percentage of the total cell count for each sample.

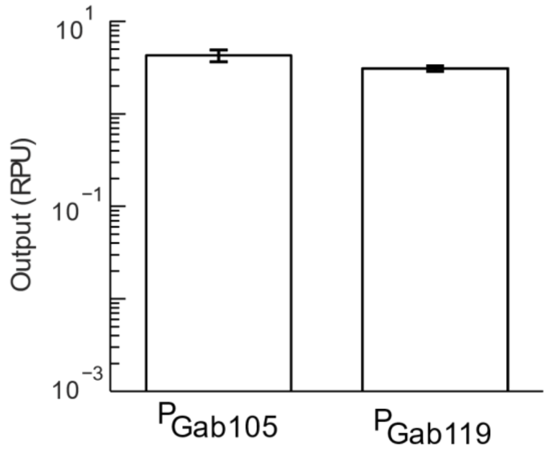

Supplementary Figure 5. Synthetic P_{Gab} promoter output in the absence of *gabR*. The fluorescent protein eYFP is expressed by P_{Gab105} or P_{Gab119} on a backbone that does not express GabR (pML3010 and pML3011). Cells were grown in M9 media containing no inducer. Cell fluorescence was measured via flow cytometry and promoter output was converted to RPU units. The bar represents the average of the measured geometric median for a population of 5,000-10,000 cells measured in three identical experiments performed on different days. The error bar represents the standard deviation (n = 3 for P_{Gab105} and n = 2 for P_{Gab119}).

Supplementary Table 1. Plasmid constructs used in this paper

Plasmid Name	Description
pAN 1717[2]	RPU standard strain expressing *eYFP* under control of J23101 on a backbone expressing *lacI*, *tetR*, *kanR* and the p15a ori[2]
pAN871[2]	Plasmid backbone containing 3 sensors (*araC*, *lacI*, *tetR*), *kanR* and p15a ori[2]
pML1001	Synthetic promoter P1 driving *eYFP* expression on the pAN871 backbone for promoter characterization
pML1002	Synthetic promoter P2 driving *eYFP* expression on the pAN871 backbone for promoter characterization
pML1003	Synthetic promoter P3 driving *eYFP* expression on the pAN871 backbone for promoter characterization
pML1004	Synthetic promoter P4 driving *eYFP* expression on the pAN871 backbone for promoter characterization
pML1005	Synthetic promoter P5 driving *eYFP* expression on the pAN871 backbone for promoter characterization
pML1006	Synthetic promoter P6 driving *eYFP* expression on the pAN871 backbone for promoter characterization
pML2000	Propionate sensor design prototyping backbone. Plasmid backbone constitutively expresses *lacI* and *tetR* as well as the resistance gene *kanR* and has a p15a ori. The plasmid expresses PrpR under the control of P_{Tet}
pML2001	Final propionate sensor construct on a plasmid containing the repressors *lacI* and *tetR* as well as the resistance gene *kanR* and the p15a ori. The plasmid expresses PrpR under the control of constitutive promoter P6
pML2002	Plasmid expressing *eYFP* under the control of P_{Prp} on the pML2000 backbone for sensor design prototyping
pML2003	Plasmid expressing *eYFP* under the control of P_{Prp} on the pML2001 backbone for characterizing the final sensor design<br><br>GenBank accession: ON959466
pML2004	Plasmid expressing *eYFP* under the control of P_{Prp} in which PrpR is expressed under its native promoter P_{prpR} from the *E. coli* genomic fragment
pML3000	GABA sensor design prototyping backbone. Plasmid backbone containing the repressors *lacI* and *tetR* as well as the resistance gene *kanR* and the p15a ori. The plasmid expresses GabR under the control of P_{Tet}
pML3001	Final GABA sensor construct on a plasmid containing the repressors *lacI* and *tetR* as well as the resistance gene *kanR* and the p15a ori. The plasmid expresses GabR under the control of constitutive promoter P2
pML3002	Plasmid expressing *eYFP* under the control of P_{Gab105} on the pML3000 backbone for sensor design prototyping
pML3003	Plasmid expressing eYFP under the control of P_{Gab119} on the pML3000 backbone
pML3004	Plasmid expressing *eYFP* under the control of P_{Gab105}-S1 on the pML3000 backbone

pML3005	Plasmid expressing *eYFP* under the control of P_{Gab105}-S2 on the pML3000 backbone
pML3006	Plasmid expressing *eYFP* under the control of P_{Gab105}-S3 on the pML3000 backbone
pML3007	Plasmid expressing *eYFP* under the control of P_{Gab105}-UE on the pML3000 backbone
pML3008	Plasmid expressing *eYFP* under the control of P_{GabTD} on the pML3000 backbone
pML3009	Plasmid expressing *eYFP* under the control of P_{Gab105} on the pML3001 backbone for characterizing the final sensor design<br><br>GenBank accession: ON959467
pML3010	Plasmid expressing *eYFP* under the control of P_{Gab105}. Backbone contains 4 sensors (*lacI, tetR, araC, luxR*)
pML3011	Plasmid expressing *eYFP* under the control of P_{Gab119}. Backbone contains 4 sensors (*lacI, tetR, araC, luxR*)
PT_pLW555[1]	Plasmid expressing *eYFP* under the control of P_{Tet}. Backbone contains 4 sensors (*lacI, tetR, araC, luxR*)[1]

Supplementary Table 2. Genetic part sequences used in this work

Part Name	Type	DNA Sequence	Source
P_{Tet}	Promoter	tactccaccgttggctttttttccctatcagtgatagagattgacatccctatcagtgatagagataatgagcac	4
J23105	Promoter	tttacggctagctcagtcctaggtactatgctagc	3
P_1	Promoter	tttatggctagctcagtcctaggtacaatgctagcctgagtccgagtgcttacagtt	This work
P_2	Promoter	tttacggctagctcagtcctaggtatagtgctagcctgagtccgagtgcttacagtt	This work
P_3	Promoter	tttacggctagctcagccctaggtattatgctagcctgagtccgagtgcttacagtt	This work
P_4	Promoter	tttatagctagctcagcccttggtacaatgctagcctgagtccgagtgcttacagtt	This work
P_5	Promoter	ttgacggctagctcagtcctaggtattgtgctagcctgagtccgagtgcttacagtt	This work
P_6	Promoter	gataagtccctaactttgacaatgcctagatcaccgtatataatgctagca	This work
P_{GabTD}	Promoter	tcctgATACCAtcaaaaagttataatTGGTACttttcatcATACCAaagagaagtcagaatgataa	5
P_{Gab119}	Promoter	tcctgATACCAtcaaaaagttataatTGGTACttgacagcATACCAagtcctaggtataatgctagc	This work
P_{Gab105}	Promoter	tcctgATACCAtcaaaaagttataatTGGTACtttacggcATACCAagtcctaggtactatgctagc	This work
P_{Gab105}-S1	Promoter	tcctgtcaaaaagttataatTGGTACtttacggcATACCAagtcctaggtactatgctagc	This work
P_{Gab105}-S2	Promoter	tcctgATACCAtcaaaaagttataatacctactttacggcATACCAagtcctaggtactatgctagc	This work
P_{Gab105}-S3	Promoter	tcctgATACCAtcaaaaagttataatTGGTACtttacggctagctcagtcctaggtactatgctagc	This work
P_{Gab105}-UE	Promoter	tcctgATACCAttggtcatgatgaacTGGTACtttacggcATACCAagtcctaggtactatgctagc	This work
P_{Prp}	Promoter	gtcttgtttcataattgttgcaatgaaacgcggtgaaacattgcctgaaacgttaactgaaacgcatatttgcggattagt<br>tcatgactttatctctaacaaattgaaattaaacatttaattttattaaggcaattgtggcacaccccttgctttgtctttatca<br>acgcaaataacaagttgataacaagctagcaggaggaattcacc	6
$P_{WT-PrpR}$	Promoter	ttgcaacaattatgaaacaagactaaa	6
PrpRrbs	RBS	cccaatattcggtttcttaactttgcggtgcgct	6
B0064	RBS	tactagagaaagaggggaaatactag	2
ML433G	RBS	taacatgttgtcaatgaggagctatcttg	This work and [7,8]
RiboJ53	Insulator	gcggtcaacgcatgtgctttgcgttctgatgagacagtgatgtcgaaaccgcctctacaaataattttgtttaa	2
RiboJ64	Insulator	aggagtcaattaatgtgcttttaattctgatgagacggtgacgtcgaaactccctctacaaataattttgtttaa	2
RiboJ	Insulator	agctgtcaccggatgtgctttccggtctgatgagtccgtgaggacgaaacagcctctacaaataattttgtttaa	2
araC	Gene	atggctgaagcgcaaaatgatcccctgctgccgggatactcgtttaatgcccatctggtggcgggtttaacgccgatt<br>gaggccaacggttatctcgatttttttatcgaccgaccgctgggaatgaaaggttatattctcaatctcaccattcgcggt<br>caggggggtggtgaaaaatcagggacgagaatttgtttgccgaccgggtgatattttgctgttcccgccaggagagatt<br>catcactacggtcgtcatccggaggctcgcgaatggtatcaccagtgggtttactttcgtccgcgcgcctactggcat<br>gaatggcttaactggccgtcaatatttgccaatacggggttctttcgcccggatgaagcgcaccagccgcatttcagc<br>gacctgtttgggcaaatcattaacgccgggcaaggggaagggcgctattcggagctgctggcgataaatctgcttga<br>gcaattgttactgcggcgcatggaagcgattaacgagtcgctccatccaccgatggataatcgggtacgcgaggctt<br>gtcagtacatcagcgatcacctggcagacagcaattttgatatcgccagcgtcgcacagcatgtttgcttgtcgccgtc<br>gcgtctgtcacatcttttccgccagcagttagggattagcgtcttaagctggcgcgaggaccaacgtatcagccaggc<br>gaagctgcttttgagcaccacccggatgcctatcgccaccgtcggtcgcaatgttggttttgacgatcaactctatttct<br>cgcgggtatttaaaaaatgcaccggggccagcccgagcgagttccgtgccggttaa	9
gabR	Gene	atggatatcacgattacactcgatcgttcagaacaagccgattatatctatcagcaaatttatcaaaagctgaaaaaaga<br>aatcctcagccgcaatctgctgccgcactcgaaggttccctccaagcgggagctggctgaaaatctcaaggtcagc<br>gtaaattcagtgaattcagcctatcagcagctgctggctgaggggtatttgtacgccattgaacgaaagggtttcttcgt<br>ggaggaactagacatgttttccgccgaggagcaccctccatttgcactgccggatgacctaaaagagattcacatcg<br>accagagcgattggatatcgttttcacacatgagttccgatacagaccattttccgatcaaaagctggttccgctgcga<br>gcaaaaagcggcctcccgctcataccgcacgctcggcgatatgtcacatccgcaagggatatatgaagtgagagc<br>ggccattacgaggctcatttccctgacgaggggtgtaaaatgcaggccggaacaaatgatcataggggcaggcac<br>acaggtgctcatgcagctgttgactgagcttttacccaaggaagccgtgtatgcgatggaggagcctggctacaggc<br>gcatgtatcagcttttgaagaatgccggaaaacaagtaaagacgatcatgctggatgaaaaaggcatgtcgattgctg<br>aaatcaccagacagcagccagatgtgctggtgaccaccccgtcgcatcagtttccgtccggaacgattatgcctgtat<br>ccagaagaattcagctgctgaactgggcagccgaggagccgcgccgatatatcattgaggacgattatgatagtga<br>attcacatatgatgtagacagtattccggcgctgcaaagcctcgaccgttttcaaaatgtcatctatatgggaaccttttc<br>aaagtcccttctccccggcttacggatcagctatatggtgttgccgcctgagctgttgagggcatacaaacagcggg<br>gctatgatctgcagacttgctcatcactcacacagctcaccctgcaggaatttatcgagtctggtgaatatcagaagca<br>tataaaaaaaatgaagcagcattataaagaaaagagagaacgcctgatcaccgctttagaagcagagttcagcgga	5

		gaggttaccgtaaaaggggcaaatgcggggctgcattttgttaccgaatttgataccaggcgcaccgaacaagacat<br>cctgtcacatgctgccgggctgcagcttgaaatattcggaatgagccgatttaacttgaaggaaaacaagcggcaaa<br>cgggcaggcctgctctcattatcggctttgcacggctgaaggaagaagatattcaggagggtgtgcagcggcttttc<br>aaagcggtttacggacataaaaaaatccccgttacaggggattga	
prpR	Gene	atggcacatccaccacggcttaatgacgacaaaccggttatctggacggtatctgtaacgcgcctgttcgagctgtttc<br>gcgatatcagcctcgagtttgatcacctggcgaacattacccctatccagcttggctttgaaaaagcggtgacctacat<br>ccacaagaaactggcaaacgaacgctgtgacgccatcatcgccgctggatctaacggcgcgtacctgaaaagccg<br>cctgtcagtgccagttattttgattaaaccgagcggctacgatgtgttacaggcactggcaaaagccggaaaactcac<br>ctcttctatcggcgttgtcacttatcaggaaactattccggcactggtggcgtttcaaaaaacctttaatttgcgcctcgat<br>caacgtagctacattaccgaagaagatgcacgcgggcagattaacgagctaaaagctaacggcaccgaagcggtg<br>gtcggcgcggggctgattaccgatctggcagaagaagccggaatgaccggaatttttatctattccgccgccaccgt<br>gcgccaggcgttcagcgatgcgctggatatgacgcgcatgtcgttacgccataacactcacgatgccacccgcaac<br>gccctgagaactcgttacgtgctgggcgatatgctcggtcaatcaccacagatggaacaagtacggcagactattttg<br>ctgtatgcccgctccagtgcagcggtgttgattgagggggaaacggggacgggcaaagagctggcggcccaggc<br>gattcatcgggaatattttgcccgccacgatgcgcgacagggcaaaaagtcgcatccgtttgttgcagtcaactgcgg<br>ggcgattgccgaatcgctgctggaagcagaactgtttggctatgaggaaggggcgtttaccggctcgcgacgcggc<br>ggtcgcgccgggctgtttgaaatcgcccacggaggtacgctgtttctcgatgagattggcgaaatgccgctgccgtt<br>gcagacccggctgctgcgggtgctggaagaaaaagaggtcacccgcgtcggcgggcatcagcctgttccggtgg<br>atgtgcgggtcattagcgccactcactgcaatctggaagaagatatgcggcaagggcagtttcgccgtgacctgtttt<br>atcggctgagtattttgcgtctgcaattgccaccactgcgcgagcgggtggcggatattctgccactggcggaaagct<br>tttgaaagtgtctctggcggcgctctccaccccgttttctgccgcattacgccagggattacaggcaagcgaaaccgt<br>gctggtgcactacgactggccgggcaatattcgtgaactgcgcaatatgatggagcgactggcgctatttttaagtgt<br>ggaaccgacgccggatttaacgccgcaatttttgcagctgctactgccggaactggcgcgcgagtcggcgaaaact<br>cccgctccacgcttactgacaccacaacaggcactggagaaatttaatggcgataaaacagcagcggcgaattattt<br>aggcatcagccggacgacgttctggcggcggctgaaaagctga	6
eYFP	Gene	atggtgagcaagggcgaggagctgttcaccggggtggtgcccatcctggtcgagctggacggcgacgtaaacgg<br>ccacaagttcagcgtgtccggcgagggcgagggcgatgccacctacggcaagctgaccctgaagttcatctgcac<br>cacaggcaagctgcccgtgccctggcccaccctcgtgaccaccttcggctacggcctgcaatgcttcgcccgctac<br>cccgaccacatgaagctgcacgacttcttcaagtccgccatgcccgaaggctacgtccaggagcgcaccatcttctt<br>caaggacgacggcaactacaagacccgcgccgaggtgaagttcgagggcgacaccctggtgaaccgcatcgag<br>ctgaagggcatcgacttcaaggaggacggcaacatcctggggcacaagctggagtacaactacaacagccacaa<br>cgtctatatcatggccgacaagcagaagaacggcatcaaggtgaacttcaagatccgccacaacatcgaggacgg<br>cagcgtgcagctcgccgaccactaccagcagaacacccccaatcggcgacggccccgtgctgctgcccgacaacc<br>actaccttagctaccagtccgccctgagcaaagaccccaacgagaagcgcgatcacatggtcctgctggagttcgt<br>gaccgccgccgggatcactctcggcatggacgagctgtacaagtaa	10
lacI	Gene	atgaaaccagtaacgttatacgatgtcgcagagtatgccggtgtctcttatcagaccgtttcccgcgtggtgaaccag<br>gccagccacgtttctgcgaaaacgcgggaaaaagtggaagcggcgatggcggagctgaattacattcccaaccgc<br>gtggcacaacaactggcgggcaaacagtcgttgctgattggcgttgccacctccagtctggccctgcacgcgccgt<br>cgcaaattgtcgcggcgattaaatctcgcgccgatcaactgggtgccagcgtggtggtgtcgatggtagaacgaag<br>cggcgtcgaagcctgtaaagcggcggtgcacaatcttctcgcgcaacgcgtcagtgggctgatcattaactatccgc<br>tggatgaccaggatgccattgctgtggaagctgcctgcactaatgttccggcgttatttcttgatgtctctgaccagaca<br>cccatcaacagtattattttctcccatgaggacggtacgcgactgggcgtggagcatctggtcgcattgggtcaccag<br>caaatcgcgctgttagcgggcccattaagttctgtctcggcgcgtctgcgtctggctggctggcataaatatctcactc<br>gcaatcaaattcagccgatagcggaacgggaaggcgactggagtgccatgtccggttttcaacaaaccatgcaaat<br>gctgaatgagggcatcgttcccactgcgatgctggttgccaacgatcagatggcgctgggcgcaatgcgcgccatt<br>accgagtccgggctgcgcgttggtgcggatatctcggtagtgggatacgacgataccgaagatagctcatgttatatc<br>ccgccgttaaccaccatcaaacaggattttcgcctgctggggcaaaccagcgtggaccgcttgctgcaactctctca<br>gggccaggcggtgaagggcaatcagctgttgccagtctcactggtgaaaagaaaaaccaccctggcgcccaatac<br>gcaaaccgcctctccccgcgcgttggccgattcattaatgcagctggcacgacaggtttcccgactggaaagcggg<br>cagtga	4
tetR	Gene	atgtccagattagataaaagtaaagtgattaacagcgcattagagctgcttaatgaggtcggaatcgaaggtttaacaa<br>cccgtaaactcgcccagaagctaggtgtagagcagcctacattgtattggcatgtaaaaaataagcgggctttgctcg<br>acgccttagccattgagatgttagataggcaccatactcacttttgccctttagaaggggaaagctggcaagattttta<br>cgtaataacgctaaaagttttagatgtgctttactaagtcatcgcgatggagcaaaagtacatttaggtacacggcctac<br>agaaaaacagtatgaaactctcgaaaatcaattagcctttttatgccaacaaggtttttcactagagaatgcattatatgc<br>actcagcgctgtggggcattttactttaggttgcgtattggaagatcaagagcatcaagtcgctaaagaagaaaggga<br>aacacctactactgatagtatgccgccattattacgacaagctatcgaattatttgatcaccaaggtgcagagccagcc<br>ttcttattcggccttgaattgatcatatgcggattagaaaaacaacttaaatgtgaaagtgggtcctaa	4
kanR	Gene	Atgagccatattcaacgggaaacgtcttgctccaggccgcgattaaattccaacatggatgctgatttatatgggtata<br>aatgggctcgcgataatgtcgggcaatcaggtgcgacaatctatcgattgtatgggaagcccgatgcgccagagttg<br>tttctgaaacatggcaaaggtagcgttgccaatgatgttacagatgagatggtcagactaaactggctgacggaattta<br>tgcctcttccgaccatcaagcattttatccgtactcctgatgatgcatggttactcaccactgcgatccccgggaaaaca<br>gcattccaggtattagaagaatatcctgattcaggtgaaaatattgttgatgcgctggcagtgttcctgcgccggttgca<br>ttcgattcctgtttgtaattgtccttttaacagcgatcgcgtatttcgtctcgctcaggcgcaatcacgaatgaataacggt<br>ttggttgatgcgagtgattttgatgacgagcgtaatggctggcctgttgaacaagtctggaaagaaatgcataagctttt<br>gccattctcaccggattcagtcgtcactcatggtgatttctcacttgataaccttatttttgacgaggggaaattaataggt<br>tgtattgatgttggacgagtcggaatcgcagaccgataccaggatcttgccatcctatggaactgcctcggtgagtttt<br>ctccttcattacagaaacggctttttcaaaaatatggtattgataatcctgatatgaataaattgcagtttcatttgatgctcg<br>atgagtttttctaa	1

L3S3P21	Terminator	ccaattattgaaggcctccctaacggggggcctttttttgtttctggtctccc	11
L3S2P21	Terminator	ctcggtaccaaattccagaaaagaggcctcccgaaaggggggcctttttttcgttttggtcc	11
L3S3P22*	Terminator	ccaattattgaaggccgctaacgcggcctttttttgtttctggtcaccc	11
L3S3P41	Terminator	aaaaaaaaaaaacaccctaacgggtgtttttttttttttggtctccc	11

* The underlined nucleotide was mutated from the original part to remove a BsaI restriction site.

References

(1) Andrews, L. B.; Nielsen, A. A. K.; Voigt, C. A. Cellular Checkpoint Control Using Programmable Sequential Logic. *Science* **2018**, *361* (6408), eaap8987. https://doi.org/10.1126/science.aap8987.

(2) Nielsen, A. A. K.; Der, B. S.; Shin, J.; Vaidyanathan, P.; Paralanov, V.; Strychalski, E. A.; Ross, D.; Densmore, D.; Voigt, C. A. Genetic Circuit Design Automation. *Science* **2016**, *352* (6281), aac7341–aac7341. https://doi.org/10.1126/science.aac7341.

(3) Kelly, J. R.; Rubin, A. J.; Davis, J. H.; Ajo-Franklin, C. M.; Cumbers, J.; Czar, M. J.; de Mora, K.; Glieberman, A. L.; Monie, D. D.; Endy, D. Measuring the Activity of BioBrick Promoters Using an in Vivo Reference Standard. *J. Biol. Eng.* **2009**, *3* (1), 4. https://doi.org/10.1186/1754-1611-3-4.

(4) Stanton, B. C.; Nielsen, A. A. K.; Tamsir, A.; Clancy, K.; Peterson, T.; Voigt, C. A. Genomic Mining of Prokaryotic Repressors for Orthogonal Logic Gates. *Nat. Chem. Biol.* **2014**, *10* (2), 99–105. https://doi.org/10.1038/nchembio.1411.

(5) Nardella, C.; Barile, A.; Salvo, M. L.; Milano, T.; Pascarella, S.; Tramonti, A.; Contestabile, R. Interaction of Bacillus Subtilis GabR with the GabTD Promoter: Role of Repeated Sequences and Effect of GABA in Transcriptional Activation. *FEBS J.* **2020**, *287* (22), 4952–4970. https://doi.org/10.1111/febs.15286.

(6) Lee, S. K.; Keasling, J. D. A Propionate-Inducible Expression System for Enteric Bacteria. *Appl. Environ. Microbiol.* **2005**, *71* (11), 6856–6862. https://doi.org/10.1128/AEM.71.11.6856-6862.2005.

(7) Cetnar, D. P.; Salis, H. M. Systematic Quantification of Sequence and Structural Determinants Controlling MRNA Stability in Bacterial Operons. *ACS Synth. Biol.* **2021**, *10* (2), 318–332. https://doi.org/10.1021/acssynbio.0c00471.

(8) Reis, A. C.; Salis, H. M. An Automated Model Test System for Systematic Development and Improvement of Gene Expression Models. *ACS Synth. Biol.* **2020**, *9* (11), 3145–3156. https://doi.org/10.1021/acssynbio.0c00394.

(9) Lee, S. K.; Chou, H. H.; Pfleger, B. F.; Newman, J. D.; Yoshikuni, Y.; Keasling, J. D. Directed Evolution of AraC for Improved Compatibility of Arabinose- and Lactose-Inducible Promoters. *Appl. Environ. Microbiol.* **2007**, *73* (18), 5711–5715. https://doi.org/10.1128/AEM.00791-07.

(10) Cormack, B. P.; Valdivia, R. H.; Falkow, S. FACS-Optimized Mutants of the Green Fluorescent Protein (GFP). *Gene* **1996**, *173* (1), 33–38. https://doi.org/10.1016/0378-1119(95)00685-0.

(11) Chen, Y.-J.; Liu, P.; Nielsen, A. A. K.; Brophy, J. A. N.; Clancy, K.; Peterson, T.; Voigt, C. A. Characterization of 582 Natural and Synthetic Terminators and Quantification of Their Design Constraints. *Nat. Methods* **2013**, *10* (7), 659–664. https://doi.org/10.1038/nmeth.2515.
